# Interplay between Light and Plant Hormones in the Control of *Arabidopsis* Seedling Chlorophyll Biosynthesis

**DOI:** 10.3389/fpls.2017.01433

**Published:** 2017-08-17

**Authors:** Xiaoqin Liu, Yue Li, Shangwei Zhong

**Affiliations:** State Key Laboratory of Protein and Plant Gene Research, School of Advanced Agricultural Sciences and School of Life Sciences, Peking University Beijing, China

**Keywords:** light signaling, plant hormones, chlorophyll biosynthesis, de-etiolation, PIFs, HY5, EIN3/EIL1, DELLAs

## Abstract

Chlorophyll biosynthesis is one of the most important cellular processes and is essential for plant photosynthesis. After germination under the soil, dark-grown seedlings are etiolated and accumulate the chlorophyll precursor protochlorophyllide (Pchlide) in cotyledons. Upon exposure to light, Pchlide is rapidly converted to chlorophyll to initiate photoautotrophic growth. In this light-regulated de-etiolation process, multiple endogenous phytohormones are also involved. Although the co-regulation of seedling greening by light and hormones has long been observed, recent studies greatly advanced our understanding of their interplay by identifying the key components connecting these pathways. The integrators, such as PHYTOCHROME-INTERACTING FACTORs, ELONGATED HYPOCOTYL 5, ETHYLENE INSENSTIVE 3 and DELLA proteins, are key transcription regulators in light or hormone signaling pathways. This review focuses on these integrators and illustrates the regulatory networks of light and hormone interactions in chlorophyll biosynthesis.

## Introduction

Crop seeds are usually buried in soil, whereby post-germinative seedlings become etiolated and grow upward heterotrophically from seed reserves. Upon reaching the soil surface, etiolated seedlings undergo a dramatic developmental transition termed de-etiolation, which includes cotyledon opening and greening (Von Arnim and Deng, 1996; Chen et al., 2004). This transition is of particular vulnerability for plant survival, requiring rapid initiation of photoautotrophic growth without causing photooxidation (Huq et al., 2004; Zhong et al., 2014). To successfully accomplish this, chlorophyll biosynthesis must be strictly controlled.

In higher plants, chlorophyll is initially biosynthesized from glutamate, which is then converted to 5-aminolevulinic acid (ALA) and further converted to protochlorophyllide (Pchlide) (Tanaka et al., 2011). When the dark-grown seedlings are exposed to light, the rate-limiting enzymes NADPH protochlorophyllide oxidoreductases (PORs) are photoactivated and catalyze the conversion of Pchlide to chlorophyllide, which is subsequently esterified to mature chlorophyll (Fujita, 1996; Reinbothe et al., 2010). In *Arabidopsis*, three POR enzymes, PORA, PORB and PORC have been identified, with PORA/PORB playing the main roles in young seedlings (Buhr et al., 2008). Because Pchlide is extremely phototoxic, its amount must be stoichiometrically matched with the level of POR enzymes. Over-accumulation of the free Pchlide that cannot be converted to chlorophyll in time will result in the production of reactive oxygen species (ROS) upon light exposure, causing photooxidative damage to the seedlings (op den Camp et al., 2003; Huq et al., 2004; Chen et al., 2013; Zhong et al., 2014). To survive, seedlings have evolved efficient ways to adjust the levels of Pchilde and POR enzymes to allow for rapid establishment of photosynthesis without causing photobleaching (op den Camp et al., 2003; Huq et al., 2004; Zhong et al., 2014). Moreover, carotenoid biosynthesis is also markedly upregulated to protect the etioplasts from photooxidative damage by quenching excess excitation energy when seedlings are exposed to light (Rodriguez-Villalon et al., 2009).

Light is the main environmental factor that regulates the pathway of chlorophyll biosynthesis, while plant hormones are also recruited to mediate the developmental switch of de-etiolation. Some key components in the light signaling pathway, such as PIFs and HY5, connect light signals to the signaling pathways of multiple phytohormones, including ethylene, gibberellin (GA) and cytokinin (CK). In this review, we concentrate on how chlorophyll biosynthesis is cooperatively regulated by light and endogenous hormone signals, focusing on the interplay between light and hormone signaling pathways during seedling de-etiolation.

### Chlorophyll Biosynthesis Regulated by Key Components in Light Signaling Pathway

Light provides plants with energy for photosynthesis and a major source of information about their environment. Both light quality and quantity are constantly monitored by plants through a group of photoreceptors (Quail, 2002; Chen et al., 2004). Among them, phytochromes (phys, including phyA-phyE in *Arabidopsis*) sense far-red and red light (Quail, 2002; Chen et al., 2004). The perception of light signals by phys initiates an intracellular transduction to alter the expression of nuclear genes (Quail, 2002; Chen et al., 2004; Leivar and Quail, 2011). There are two groups of transcription factors, PIFs and HY5, that mediate light-induced responses in opposite ways (Von Arnim and Deng, 1996; Chen et al., 2004; Leivar and Quail, 2011). PIFs are negative regulators and are directly targeted by photoactivated phys for degradation (Ni et al., 1998, 2014; Huq et al., 2004; Shen et al., 2008), while HY5 is stabilized by light to promote photomorphogenesis (Ang et al., 1998; Osterlund et al., 2000). During the process of de-etiolation, both PIFs and HY5 have been shown to transcriptionally regulate the chlorophyll biosynthesis pathway.

PHYTOCHROME-INTERACTING FACTORs accumulate in dark-grown seedlings and negatively regulate the tetrapyrrole metabolism of chlorophyll biosynthesis (Huq et al., 2004; Monte et al., 2004; Moon et al., 2008; Shin et al., 2009; Stephenson et al., 2009). Mutation of *PIF1* and *PIF3* results in an excessive amount of Pchlide in the dark and causes severe photobleaching upon light exposure (Huq et al., 2004; Monte et al., 2004; Moon et al., 2008; Shin et al., 2009; Stephenson et al., 2009). Further experiments reveal that PIF1 directly binds to the promoter of *PORC*, while PIF3 represses the expression of *HEMA1, GUN4* and *CHLH* genes (Moon et al., 2008; Stephenson et al., 2009). HEMA1 is the main glutamyl-tRNA reductase that catalyzes the rate-limiting step for ALA biosynthesis, while GUN4 and CHLH promote the conversion of ALA to the chlorophyll biosynthetic branch (Stephenson and Terry, 2008; Tanaka et al., 2011). In addition, PIF5 has been shown to be involved in the negative regulation of *CHLH* gene expression in etiolated seedlings (Shin et al., 2009), and a large portion of nuclear-encoded chlorophyll biosynthesis genes are notably upregulated in the *pifQ* mutant (lacking *PIF1, PIF3, PIF4* and *PIF5* genes) (Leivar et al., 2009; Shin et al., 2009). PIF1 was also found to partly repress the transposase-derived transcription factor FHY3/FAR1-activated gene expression of *HEMB1* that encodes the ALA dehydratase (Tang et al., 2012), and chromatin-remodeling enzyme BRM interacts with PIF1 to modulate *PORC* expression (Zhang et al., 2017). Moreover, PIFs have been reported to directly repress the gene expression of *PSY* (phytoene synthase), which is the main rate-determining enzyme of carotenoid biosynthesis (Toledo-Ortiz et al., 2010). When PIFs are degraded by light, carotenoids are rapidly synthesized to coordinate with chlorophyll biosynthesis, thus facilitating the assembly of functional photosynthetic machinery (Toledo-Ortiz et al., 2010). Therefore, PIFs play important roles in the fine tuning of tetrapyrrole metabolism, directly or indirectly regulating chlorophyll biosynthesis and photosynthetic genes to optimize the seedling greening process.

ELONGATED HYPOCOTYL 5 functions downstream of the photoreceptors and central repressors in the light signaling pathway to promote seedling photomorphogenesis. In the dark, HY5 is degraded through the COP1/DET1-mediated ubiquitination degradation pathway (Ang et al., 1998; Osterlund et al., 2000). HY5 plays a vital role in the convergence of blue, red and far-red light-signal pathways for regulating the transcription levels of *HEMA1* (McCormac and Terry, 2002). Several nuclear-encoding photosynthetic and chlorophyll biosynthesis genes, such as *CHLH, GUN4, PORC, CAO* and *CHL27*, are the putative targets of HY5 (Lee et al., 2007). Although roots are heterotrophic organs, lots of chlorophyll accumulates in light-grown *det1* and *cop1* mutant roots, and HY5 mediates the process of chlorophyll synthesis in roots (Chory and Peto, 1990; Deng et al., 1992; Ang et al., 1998). In addition, a Myb-like transcription factor REVEILLE1 (RVE1) was recently found to act downstream of phyB to modulate chlorophyll biosynthesis by directly activating *PORA* expression (Xu et al., 2015; Jiang et al., 2016).

### Ethylene Is Crucial for Cotyledon Greening and Survival of Seedling Soil Emergence

Plant hormones are small molecules that mediate a myriad of cellular responses. Many hormones are involved in light-induced seedling greening. One prominent factor affecting chlorophyll biosynthesis is ethylene, which dramatically represses Pchlide accumulation and induces the gene expression of both *PORA* and *PORB* in etiolated seedlings (Zhong et al., 2009, 2010, 2014). Thus, ethylene plays a critical role in protecting cotyledons from photooxidative damage when the seedlings are exposed to light. The effects of ethylene are mediated by EIN3/EIL1, the master transcription factors in the ethylene signaling pathway (Chao et al., 1997; Guo and Ecker, 2004). EIN3/EIL1 markedly repress the accumulation of Pchlide and directly bind to the promoters of *PORA* and *PORB* to activate their gene expression (Zhong et al., 2009, 2010, 2014). Genetic studies reveal that EIN3/EIL1 cooperate with PIF1 and act downstream of COP1 in promoting seedling greening (Zhong et al., 2009). The protein levels of EIN3 are enhanced by COP1 but are decreased by light (Zhong et al., 2009; Shi et al., 2016a,b). In addition, overexpressing EIN3 rescues the far-red light-triggered cotyledon greening defects (Zhong et al., 2009).

After germination in soil, the mechanical impedance of soil boosts ethylene production to adjust seedling morphogenesis to enhance the lifting capacity and protect against mechanical injuries (Zhong et al., 2014; Shen et al., 2016; Shi et al., 2016a). EIN3/EIL1 directly activate two independent pathways, an ERF1 pathway to slow down cell elongation and a PIF3 pathway to control Pchlide biosynthesis (Zhong et al., 2012, 2014). These two pathways are coupled to maintain a suitable amount of Pchilde to rapidly initiate photoautotrophic growth without causing photooxidation upon emergence (Zhong et al., 2014). When seedlings penetrate their way toward the surface, the dim light under the soil increases gradually and represses COP1 protein activity (Shi et al., 2016a). COP1 has been found to be the E3 ligase of EBF1 and EBF2, the F-box proteins of the E3 ligases for EIN3 degradation (Shi et al., 2016a). Therefore, COP1 and ethylene mediate the soil-imposed light and mechanical stress signals, respectively, to adjust EIN3 protein levels in response to soil condition changes when seedlings grow upward in the soil (Shi et al., 2016a). Interestingly, EIN3 also promotes the nuclear enrichment of COP1 protein to generate a positive feedback for EIN3 stability regulation (Yu et al., 2013, 2016). At the moment of emergence and reaching sunlight, photoactivated photoreceptor phyB directly interacts with EIN3 and rapidly degrades EIN3 by bringing it to the E3 ligases EBF1 and EBF2 (Shi et al., 2016b). As a result, the repression of photomorphogenesis by EIN3 and ethylene is rapidly lifted to initiate de-etiolation effectively.

### Gibberellin Regulates Chlorophyll Biogenesis Partially via the Light Signaling Pathway

Seedling de-etiolation is also subject to gibberellin (GA) regulation, as inhibiting gibberellin signaling can induce partial photomorphogenesis in the dark (Alabadi et al., 2004, 2008). DELLAs are a subfamily of the GRAS transcriptional regulators and negatively regulate gibberellin signaling to repress GA-mediated responses (Jiang and Fu, 2007). Moreover, DELLAs inhibit the transcription activity of PIF3 and PIF4 through direct blocking of the DNA-recognition domain of these factors (de Lucas et al., 2008; Feng et al., 2008). In dark-grown seedlings, DELLAs accumulate and regulate the biosynthetic pathways of both carotenoid and chlorophyll (Cheminant et al., 2011). DELLAs upregulate the expression of genes involved in chlorophyll biosynthesis (*CHLH, PORC* and *CAO*) and photosynthesis (*LHCB2.2, PSAG* and *PSAE-1*) in a PIF-dependent manner (Cheminant et al., 2011). In addition, DELLAs also positively regulate *PORA* and *PORB* gene expression independently of PIFs and repress ROS-induced photooxidative damage during de-etiolation (Cheminant et al., 2011). However, the regulation of HY5 on gibberellin-mediated chlorophyll biosynthesis seems more moderate than that of PIFs in dark conditions (Cheminant et al., 2011).

### Cytokinin Plays an Important Role in Chlorophyll Biosynthesis and Chloroplast Development

Exogenous cytokinin treatment induces cotyledon expansion and chloroplast partial differentiation (Chory et al., 1994; Vandenbussche et al., 2007). Two GATA family transcription factors, GNC and CGA1/GNL, are induced by cytokinin and regulate the expression of many chloroplast-related genes (Hudson et al., 2011; Chiang et al., 2012). Dark-grown seedlings display small etioplasts with prolamellar bodies in the absence of cytokinin, while large lens-shaped plastids contain some prothylakoid membranes in the presence of cytokinin (Chory et al., 1994). Recent reports indicate that cytokinin mediates the etioplast-to-chloroplast transition by promoting characteristic ultrastructural changes (Cortleven and Schmulling, 2015; Cortleven et al., 2016). Cytokinin signal is perceived by the receptors AHK2 and AHK3 and transduced to B-type ARR transcription factors (Argyros et al., 2008). ARRs directly regulate the expression of genes in chlorophyll biosynthesis and the light harvesting complex, such as *HEMA1* and *LHCB6* (Cortleven and Schmulling, 2015; Cortleven et al., 2016). As cytokinin has been reported to increase the protein levels of HY5 (Vandenbussche et al., 2007), it is possible that HY5 is a point of convergence between light and cytokinin signaling pathways.

### The Function of Other Plant Hormones in Regulating Seedling Greening

In addition to the well-documented hormones just described, other hormones are also important in regulating seedling greening. Auxin represses HY5 protein accumulation via IAA14 and its regulatory target ARFs in roots (Kobayashi et al., 2012). Moreover, chlorophyll synthesis genes are markedly activated in detached roots via cytokinin but are repressed by auxin (Kobayashi et al., 2017), suggesting that auxin signaling is also involved in the regulation of chlorophyll biosynthesis in the root greening response. However, further analyses are required to elucidate the regulatory network of auxin and light signals in regulating chlorophyll biosynthesis. Brassinosteroid (BR) is known to be involved in the process of de-etiolation. Many chlorophyll biosynthesis genes are upregulated from the microarray data of BR-insensitive bri1-116 seedlings in darkness (Sun et al., 2010). The key transcriptional factor GATA2 has been identified in mediating the crosstalk between BR and light signaling pathways (Luo et al., 2010). Recently, ABI4 was found to activate *COP1* expression to repress seedling de-etiolation (Xu et al., 2016). In addition, strigolactones are reported to also be involved in light signaling via regulating the nuclear localization of COP1 (Tsuchiya et al., 2010), and jasmonate inhibits COP1 activity to promote photomorphogenesis (Zheng et al., 2017). However, the signaling pathway of ABA, strigolactones and jasmonate in regulating chlorophyll biosynthesis remains largely unknown.

## Conclusion and Perspectives

**FIGURE 1:**
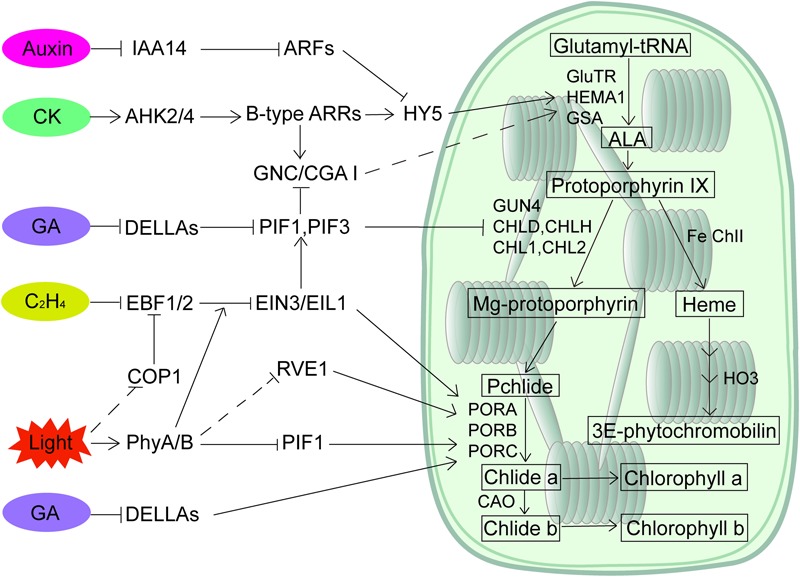
A regulatory network of chlorophyll biosynthesis by light and plant hormones. PIFs and HY5 are major transcription factors in mediating light-regulated chlorophyll biosynthesis. Multiple hormones participate in chlorophyll biosynthesis through both transcriptional and post-transcriptional regulation of PIFs and HY5, while light regulates the action of key components such as EIN3/EIL1 in hormone signaling pathways to modulate the hormonal responses.

Involvement of plant hormones in light-regulated seedling greening has been known for decades. However, we have not identified the molecular links connecting light signaling to the multiple hormonal pathways until recent years. The key transcription factors of both light and hormone signaling pathways appear to be the integrators (**Figure 1**). EIN3 directly activates the gene expression of *PORA*/*PORB* and represses Pchlide accumulation to optimize the greening process. The repression of EIN3 in synthesizing Pchlide is through activating *PIF3* transcription, whereas both phyB and COP1 predominantly regulate the protein levels of EIN3. PIFs play a pivotal role in integrating light and GA signals, and DELLAs directly sequester the transcription activity of PIFs. In addition, HY5 protein stability is regulated by auxin and cytokinin to coordinate these signals in mediating root greening, while COP1 could be new integrator as its nuclear localization can be regulated by ethylene, strigolactone and jasmonate hormones. Further studies, such as identifying additional integrators in light and hormonal signaling pathways and addressing how these components are integrated in regulating seedling greening, are needed. Moreover, we are only beginning to address the regulation of chloroplast development. Whether and how plant hormones regulate the etioplast-chloroplast differentiation process is critical in filling the gaps of greening. In summary, although we have not obtained a detailed network depicting how seedling greening is regulated by light and all the hormonal signals, the identification of key transcription regulators as signaling integrators has created a great starting point.

## Author Contributions

SZ proposed the topic. SZ, XL, and YL collected the literature and critically assessed the information. XL and SZ wrote the manuscript.

## Conflict of Interest Statement

The authors declare that the research was conducted in the absence of any commercial or financial relationships that could be construed as a potential conflict of interest.
